# The Influence of Rurality on Fruit and Vegetable Intake and BMI: Findings in Mississippi Are Not Consistent with Those at the National Level

**DOI:** 10.3390/ijerph18095021

**Published:** 2021-05-10

**Authors:** Danielle Fastring, Danielle Nadorff, Hilary DeShong

**Affiliations:** 1 College of Osteopathic Medicine, William Carey University, Hattiesburg, MS 39401, USA; 2 Department of Psychology, Mississippi State University, Starkville, MS 39762, USA; Danielle.Nadorff@msstate.edu (D.N.); hld166@msstate.edu (H.D.)

**Keywords:** nutrition, rurality, fruit and vegetable consumption

## Abstract

Sixty percent of Americans have at least one chronic disease that is both diet-related and preventable. Those living in rural areas often experience a greater burden of disease than those who live near a city center. The purpose of this study is to determine the influence of rurality on fruit and vegetable (FV) consumption and BMI. Additionally, the study compares national results to those in Mississippi, a state with an aging population, and high rates of poverty, rurality, poor diet, and obesity. Data utilized were from the 2017 Behavioral Risk Factor Surveillance System. One-way analyses of covariance were performed to determine impact of rurality on nutritional intake and BMI, while controlling for age, income, education, race, and the presence of children in the home. At the national level, rurality had a significant impact on BMI, and the daily intake of fruit juice, fruits, dark green vegetables, French fries, potatoes, other vegetables, and total daily vegetable intake. BMI and nutritional intake of those living in Mississippi was significantly poorer than those living in other states. More research is needed to determine how to best facilitate access to healthy FVs for those living in rural communities.

## 1. Introduction

A chronic condition is defined as “any physical or mental health condition that lasts more than one year and causes functional restrictions or requires ongoing monitoring or treatment” [1,2,3]. In the United States, chronic diseases are among the most common health conditions and are among the most costly to treat and manage [4]. Approximately sixty percent of all Americans have one or more chronic diseases that are considered to be both diet-related and preventable [5,6]. Diet-related chronic diseases include, but are not limited to, obesity, diabetes, cardiovascular disease, cancer, osteoporosis and bone fractures, and dental disease [7].

In a prospective cohort study which analyzed data from the first National Health and Nutrition Examination Survey Epidemiologic Follow-up Study [8,9], findings show that after adjustment for covariates such as age, race, sex, history of diabetes, physical activity, education level, regular alcohol consumption, current smoking, vitamin supplement use, and total energy intake, intake of fruit and vegetables at least three times per day was associated with a 15% lower mortality from all causes (0.85; 0.72, 1.00; P for trend = 0.02), a 24% lower mortality from ischemic heart disease (0.76; 0.56, 1.03; P for trend = 0.07), a 27% lower mortality from cardiovascular disease (0.73; 0.58, 0.92; P for trend = 0.008), a 42% lower mortality from stroke (0.58; 0.33, 1.02; P for trend = 0.05), and 27% lower incidence of stroke (RR: 0.73; 95% CI: 0.57, 0.95; P for trend = 0.01), when compared with intake of fruit and vegetables less than one time per day. In a second prospective cohort study which examined the relationship between the quantity of fruit and vegetables consumed and incident cardiovascular disease among women participating in the Nurses’ Health Study and men participating in the Health Professionals Follow-Up Study, findings show that participants in the highest quintile for fruit and vegetable consumption had a 17% lower risk (95% CI: 9%, 24%) of Coronary Heart Disease [9,10]. 

To improve outcomes associated with diet-related chronic diseases, the *2015–2020 Dietary Guidelines for Americans* developed five broad guidelines. They recommend “a healthy eating pattern across the lifespan; a diet that focuses on variety, nutrient density, and amount; limiting calories from added sugars and saturated fats and reducing sodium intake; shifting to healthier food and beverage choices; and supporting healthy eating patterns for all.” More specifically, a healthy eating pattern consists of a diet rich in whole fruits and a wide variety of vegetables from all subgroups [6]. Factors associated with disparities in fruit and vegetable consumption have been widely reported. Differences exist with regard to demographics such as age [11,12,13], race and ethnicity [14,15,16,17,18], and socioeconomic factors [19,20,21]. 

Mississippi consistently has the most inferior health outcomes of any state in the US. More specifically, Mississippi residents experience one of the highest burdens of diet-related chronic disease in the nation. Mississippi continually has the highest cardiovascular death rate of any other state in the country, with 363.2 per 100,000 deaths attributed to cardiovascular disease annually [9]. They also have the highest prevalence of adult obesity in the nation (39.5%). According to the Mississippi Obesity Action plan [22], approximately 37% of the Mississippi adult population age 20 and older have a body mass index (BMI) greater than or equal to 30 kg/m^2^. Mississippi has the third-highest prevalence of Type 2 Diabetes Mellitus in the US (14.3%). Hip fracture among those age 65 and older is a marker for osteoporosis, whereas the national average of hospitalizations for hip fracture among Medicare enrollees ages 65 and older is 5.9 per 1000 population, Mississippi ranks 45th in the nation with a rate of 7.3 per 1000 population [9].

The average vegetable consumption in the US ranges from 1.8 to 3.1 vegetables per day, with Mississippians consuming on average 1.8 vegetables per day [9]. The Centers for Disease Control and Prevention report that only 8.7% of Mississippi adults meet the daily fruit intake recommendations, and only 6.2% of Mississippi adults meet the daily vegetable intake recommendations outlined in the 2015–2020 Dietary Guidelines for America [6,23]. 

Approximately 70 million people (23%) live in geographical locations that are classified as rural areas [24]. Rurality can further impact diet-related chronic disease outcomes. Rurality has been associated with a greater risk of all-cause mortality and higher rates of diet-related chronic disease [25]. Mortality rates for diet-related causes of death (cardiovascular disease, cancer, and stroke) have been higher in non-metropolitan areas than in metropolitan areas throughout the last decade [26]. The prevalence of obesity is also disproportionately higher in rural areas [27,28]. Lastly, living in a rural location is associated with poor dietary habits such as consuming too few fruits and vegetables [29]. 

Poverty and age must also be considered as factors impacting fruit and vegetable consumption. Having fewer resources to purchase fruits and vegetables reduces consumption. Data from the 2015 BRFSS [23] indicated that 7.0% of adults living in poverty met the recommended daily allowance for vegetable intake, compared to 11.4% of adults in the highest household income category. Though not as disparate, 11.9% of adults living in poverty met the recommended daily allowance for fruit intake, compared to 13.0% of adults in the highest household income category. In the 2015 State of the Plate Report [30], data show that, since 2009, there have been double-digit losses in fruit (−11%) and vegetable (−12%) consumption among middle-aged and older individuals. 

### 1.1. Current Study

The purpose of the current study was to determine the influence of rurality on fruit and vegetable consumption and BMI within a nationwide sample when controlling for age, income level, education, race, and presence of children in the home. A second aim of the current study was to compare national results with those specific to participants living in Mississippi, a state known for its aging population [24], poverty [31], high percentage of rurality [24], poor diet [9], and prevalence of obesity (BMI ≥ 30) [9] among its populace.

### 1.2. Hypotheses

It was hypothesized that, on a national level, rurality would be negatively associated with recommended nutritional intake after controlling for age, income level, education, race, and presence of children, such that the further participants are located from a city center within an MSA, the less likely they would be to report consumption of recommended nutrition levels. Specifically, people further from an MSA are hypothesized to consume less fruit juice per day (H1), fewer dark green vegetables per day (H2), fewer servings of other types of vegetables (H3), less total fruits per day (H4), and less total vegetables per day (H5). 

It was similarly hypothesized that on a national level, rurality would be positively associated with poor nutritional intake after controlling for age, income level, education, race, and presence of children, such that the further participants are located from a city center within an MSA, the more likely they would be to report consuming food that is recommended to only eat in sparse quantities. Specifically, people further from an MSA are hypothesized to eat more french fries per day (H6) and to eat more potatoes per day (H7).

It was hypothesized that nationwide, rurality would be positively associated with computed BMI after controlling for age, income level, education, race, and presence of children, such that the further participants are located from a city center within an MSA, the higher their computed BMI would be (H8).

Next, these same hypotheses were tested within a sample of those living in Mississippi, with the same expected directionality as in the nationwide sample (H9–H16). Lastly, it was hypothesized that the nutritional intake and BMI of those living in Mississippi would be significantly poorer than those living in the rest of the United States. Specifically, residents of Mississippi were hypothesized to report significantly lower levels of fruit juice intake (H17), dark green vegetable intake (H18), other vegetable intake (H19), total fruit intake (H20), and total vegetable intake (H21) than those living in other states, after controlling for age, income, race, education, and the presence of children in the home. They were hypothesized to have significantly higher levels of french fry intake (H22) and potato intake (H23), as well as significantly higher computed BMIs (H24) than those living in other states after controlling for age, income, race, education, and the presence of children in the home.

## 2. Materials and Methods

The study utilized data collected from the 2017 Behavioral Risk Factor Surveillance System (BRFSS) [32]. The BRFSS is a CDC-sponsored yearly survey of the health-related risks, behaviors, health conditions, and preventative behaviors of a nationally representative sample consisting of more than 400,000 community-dwelling participants. It is a cross-sectional telephone survey conducted by state departments of health over “landline and cellular telephones with a standardized questionnaire and methodological assistance from the CDC.” The CDC aggregates data for each state. When conducting the landline telephone survey, data are collected from a randomly selected adult in a household. When conducting the survey via cellular phone, surveyors collect data from an adult who answers the phone after verifying that they reside in a private residence or in college housing. To be eligible for participation, respondents must be 18 years of age or older [33]. 

Each year the states participating in the BRFSS agree on the content of the questionnaire. New questions are subject to cognitive testing and field testing prior to being included. The questionnaire has three parts:

Core Component: a standard set of questions that all states use that collects data regarding current health-related perceptions, conditions, and behaviors, and demographic information.

Optional BRFSS Modules: sets of questions on specific topics that states can elect to use on their questionnaires.

State-added questions: questions developed by individual states. These questions are not edited or evaluated by the CDC.

Variables of interest included age, income level, education level, race, number of children in the home, rurality, multiple variables representing nutritional intake, and BMI. The exact age of participants was not reported within the BRFSS. Instead, there are 13 response categories, ranging from 18–24 to 80 or older. Income levels were also reported categorically, using a 5-point response scale wherein 1 = less than USD 15,000 per year and 5 = USD 50,000 or more. Height and weight were reported by the respondent. Rurality was coded upon a 4-point response scale, with the options “in the center city of an MSA (metropolitan statistical area),” “outside the center city of an MSA but inside the county containing the center city,” “inside a suburban county of the MSA,” and “not in an MSA.” Individuals’ nutritional intake was assessed via the questions in Table 1.

Participants’ response frequencies to these questions were coded in the data by the original BRFSS team as the number of daily fruit or vegetable types consumed per day, with two implied decimals (that is, a value of 124 represents an average 1.24 of this type consumed per day). BMI was calculated by the original BRFSS research team using the following standardized formula, and is reported with an implied two decimals:

[Weight (lbs)/[Height (inches)^2^ × Height (inches)]] × 703^2^ (1)

### Statistical Analysis Plan

Participant demographic variables were analyzed for frequency and percent per category. One-way analyses of covariance were performed using SPSS version 26 for Mac [34] to determine statistically significant differences on the dependent variables of nutritional intake and BMI, with the independent variable of rurality, and controlling for age, income, race, education, and presence of children in the home. Although all of our primary analyses tested hypotheses that were theory-guided or based on previous findings in the literature, which reduces the risk of spurious findings, the large number (24) of hypotheses could lead to a higher percentage of Type 1 errors. To help control for this, Bonferroni corrections using SPSS that multiply the *p* value by the number of post hoc comparisons were utilized for all pairwise comparison analyses to control for possible Type 1 errors due to multiple comparisons [35]. Additionally, a Benjamini–Hochberg procedure using the recommended false discovery rate of 25% was utilized in all main analyses to help guard against both Type 1 and Type 2 errors [36].

## 3. Results

Participants were 450,016 individuals ranging in age from 18 to over 80 years old. More than half of the sample (55.8%) were female, and the majority of respondents (51.4%) reported an annual income of less than USD 50,000. Participant demographic characteristics are reported in Table 2.

**Figure 1:**
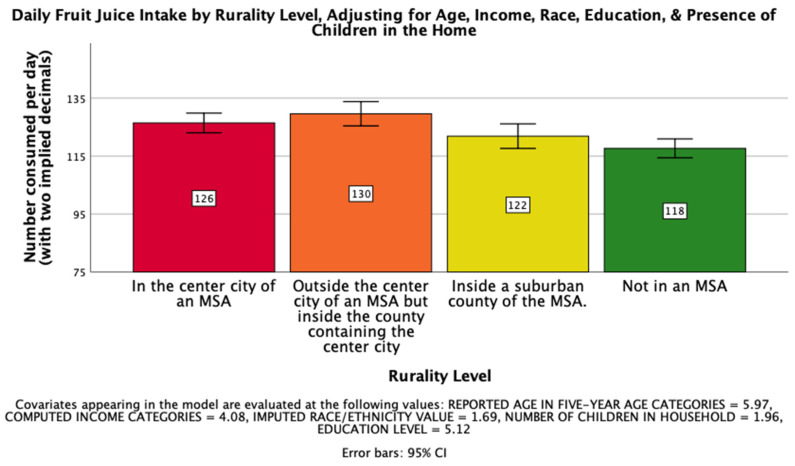
National Mean Daily Fruit Juice Intake.

First, there was a significant effect of rurality level on overall fruit and vegetable intake after controlling for age, income, race, education, and the presence of children in the home, such that those from more urban areas tended to consume fruits and vegetables more frequently *F*(3, 25202) = 3.29, *p* < 0.05, η_p_^2^ = 0.000. The first hypothesis, that rurality would have a significant impact on the daily intake of fruit juice on a national level such that those living further from an MSA would drink less, was supported. There was a significant effect of rurality level on daily fruit juice intake after controlling for age, income, race, education, and the presence of children in the home, *F*(3, 26019) = 7.834, *p* < 0.001, η_p_^2^ = 0.001. Post hoc Bonferroni pairwise comparison means with error bars representing a 95% confidence interval are presented in Figure 1. 

Post hoc Bonferroni pairwise comparison means with error bars representing a 95% confidence interval are presented in Figure 1. 

**Figure 2:**
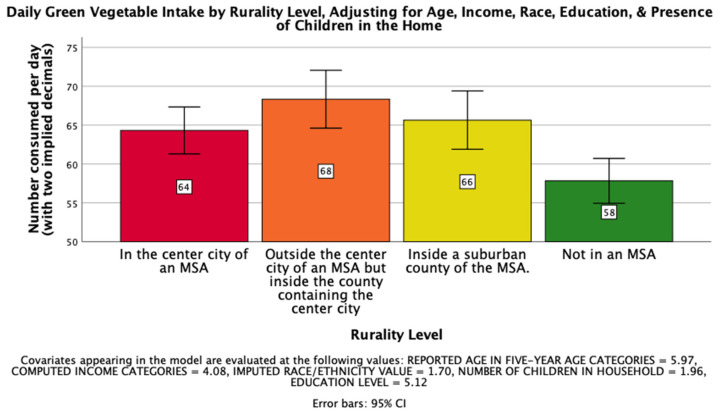
National Mean Daily Dark Green Vegetable Intake.

The second hypothesis, that rurality would have a significant impact on the daily intake of dark green vegetables on a national level such that those living further from an MSA would consume less per day, was supported. There was a significant effect of rurality level on daily dark green vegetable intake after controlling for age, income, race, education, and the presence of children in the home, *F*(3, 26023) = 7.463, *p* < 0.001, η_p_^2^ = 0.001. Post hoc Bonferroni pairwise comparison means with error bars representing a 95% confidence interval are presented in Figure 2. 

The third hypothesis, that rurality would have a significant impact on the daily intake of other vegetables on a national level such that those living further from an MSA would consume less per day, was supported. There was a significant effect of rurality level on the intake of other vegetables after controlling for age, income, race, education, and the presence of children in the home.

**Figure 3:**
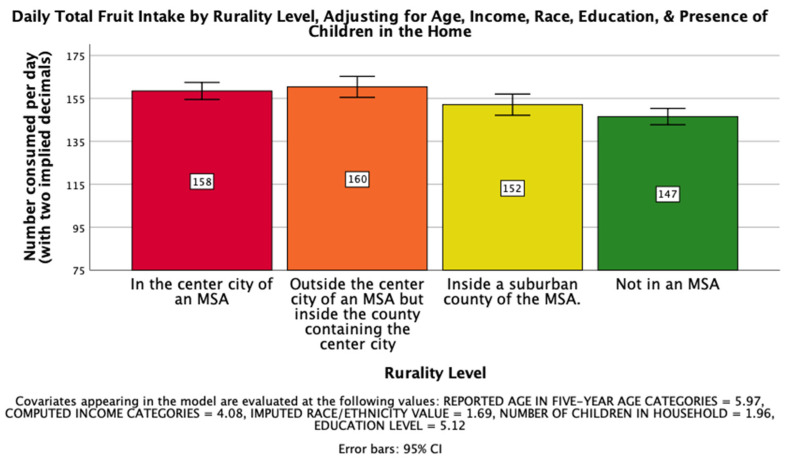
National Mean Overall Daily Fruit Intake.

The fourth hypothesis, that rurality would have a significant impact on total daily fruit intake on a national level such that those living further from an MSA would consume less fruit per day, was supported. There was a significant effect of rurality level on total daily fruit intake after controlling for age, income, race, education, and the presence of children in the home, *F*(3, 25766) = 8.819, *p* < 0.001, η_p_^2^ = 0.001. Post hoc Bonferroni pairwise comparison means with error bars representing a 95% confidence interval are presented in Figure 3. 

The fifth hypothesis, that rurality would have a significant impact on total daily vegetable intake on a national level such that those living further from an MSA would consume less vegetables per day, was not supported. There was no significant effect of rurality level on the overall daily intake of vegetables after controlling for age, income, race, education, and the presence of children in the home.

**Figure 4:**
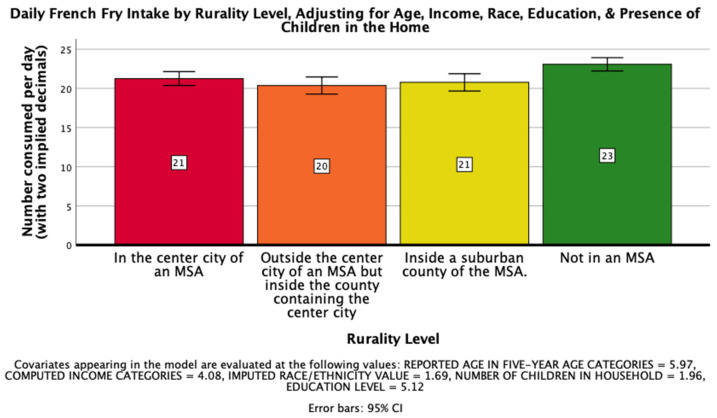
National Mean Daily French Fry Intake.

The sixth hypothesis, that rurality would have a significant impact on daily consumption of french fries on a national level such that those living further from an MSA would consume more french fries per day, was supported. There was a significant effect of rurality level on daily french fry intake after controlling for age, income, race, education, and the presence of children in the home, *F*(3, 25968) = 6.322, *p* < 0.001, η_p_^2^ = 0.001. Post hoc Bonferroni pairwise comparison means with error bars representing a 95% confidence interval are presented in Figure 4.

**Figure 5:**
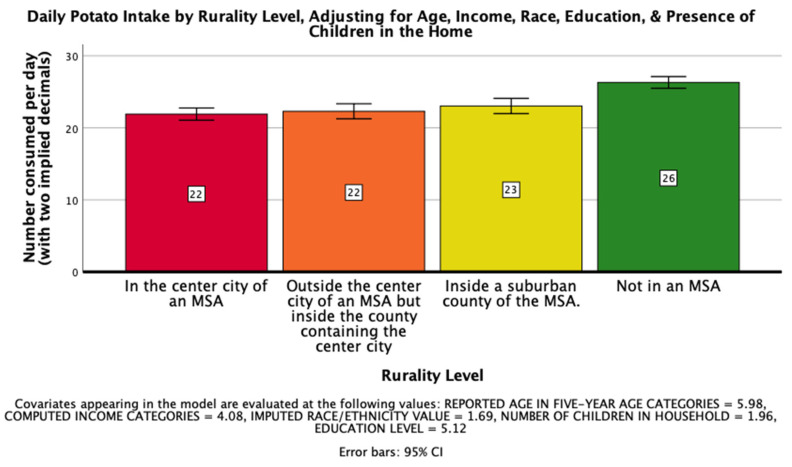
National Mean Daily Potato Intake.

The seventh hypothesis, that rurality would have a significant impact on daily consumption of potatoes on a national level such that those living further from an MSA would consume more potatoes per day, was supported. There was a significant effect of rurality level on daily potato intake after controlling for age, income, race, education, and the presence of children in the home, *F*(3, 25879) = 21.471, *p* < 0.001, η_p_^2^ = 0.002. Post hoc Bonferroni pairwise comparison means with error bars representing a 95% confidence interval are presented in Figure 5.

**Figure 6:**
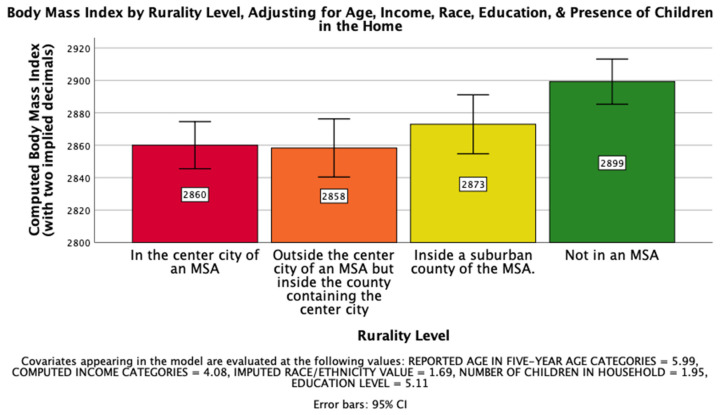
National Mean Computed Body Mass Index.

The eighth hypothesis, that rurality would have a significant impact on computed Body Mass Index on a national level such that those living further from an MSA would have a higher BMI, was supported. There was a significant effect of rurality level on computed BMI after controlling for age, income, race, education, and the presence of children in the home, *F*(3, 25800) = 6.258, *p* < 0.001, η_p_^2^ = 0.001. Post hoc Bonferroni pairwise comparison means with error bars representing a 95% confidence interval are presented in Figure 6.

Hypotheses 9–16, that the areas of nutrition and BMI hypothesized above would be replicated in a Mississippi sample after controlling for age, income, race, education, and the presence of children in the home, such that those living further from an MSA would have worse nutrition and a higher BMI than those living closer to an MSA were not supported.

Hypotheses 17–24, that after controlling for age, income, race, education, and the presence of children in the home, the nutritional intake and BMI of those living in Mississippi would be significantly poorer than those living in the rest of the United States, was supported in all but one area. The only variable in which residents of Mississippi did not significantly differ from those living in other U.S. states after controlling for age, income, race, education, and the presence of children in the home was in their daily potato consumption. Results of the one-way analyses of covariance are reported in Table 3.

## 4. Discussion

The current study analyzed several indicators of fruit and vegetable consumption at the national level and the state level within Mississippi. Specifically, the study found that at the national level, rurality had a significant impact on several variables, finding the following: those living further from an MSA drank fewer fruit juices, consumed less dark green vegetables, consumed less fruits and vegetables per day, consumed more french fries daily, consumed more potatoes per day, and had a significantly higher BMI. None of these findings were replicated in the Mississippi data. Furthermore, when comparing Mississippi to the rest of the United States, the state showed a significantly poorer rate of overall vegetable and fruit consumption, less french fry consumption, and higher rates of BMI. Overall, this study provided an updated snapshot of Mississippi’s fruit and vegetable consumption habits and BMI in comparison to the nation as a whole.

Nationally, the results demonstrate that rurality is connected with lower rates of vegetable and fruit consumption. This is likely related to a variety of factors. For instance, recent research has demonstrated that individuals in more rural environments tend to shop at small grocery stores and supercenters compared to individuals in urban and semiurban locations [37]. This is important information for potential outreach programs, as ease of access to fruits and vegetables in rural areas may be more difficult than in highly populated areas for various reasons and, therefore, the outreach programs may need to adapt in order to be more effective. For instance, a recent pilot study [38] was able to increase fruit and vegetable intake by combining an online fruit and vegetable market with online family cooking class in upstate New York—a highly rural area. 

Notably, the findings of the current study did not replicate when assessed within the state of Mississippi, a state plagued by some of the poorest health outcomes [9,22]. Future research would benefit from investigating these differences in fruit and vegetable consumption and rurality within specific states of interest. Furthermore, given the potential differences across the states, it is imperative that any kind of health outreach program designed to increase fruit and vegetable intake take into consideration potential obstacles that may be unique to that specific population or area. 

Strengths of the study include the use of data collected from a large and recent nationally representative sample of adults. This provides a strong sample from which the results can be generalized to the US population. Furthermore, with such a large sample, we were able to make comparisons between Mississippi and the US broadly, providing a unique comparison that has not previously been done. The main limitation of the current study is the use of self-reported data, as individuals may under or over report their intake of specific foods, and their height and weight due to recall bias or to social desirability of responses. A second limitation of the current study is that, with the BRFSS data set, MSA is used as the measurement for rurality, which is not an exact measure of rurality but is often stable over time and is familiar to law and policymakers. Third, the BRFSS only measures the frequency of consumption, and not the amount of the items consumed. Further research is needed to investigate specific amounts of fruit and vegetable consumption as well as the alignment with recommended daily allotments. Fourth, while having such a large and representative survey enables generalization, it does hinder our ability to take a more in-depth look into the variables of interest within the study or to truly control any variables experimentally. A smaller, more controlled study would provide this opportunity. Finally, while BMI was investigated in the current study in addition to fruit and vegetable consumption, it is noteworthy that many other factors contribute to BMI (e.g., calorie intake, physical activity) that were not included within the current study. 

There may be several reasons that explain why data from Mississippi respondents did not show the same results as those found at the national level. The state may be more homogenous with regard to nutrition intake and BMI based across all geographic designations. This theory was supported by the state-level analyses in the current study that indicated that individuals living throughout Mississippi demonstrated significantly poorer nutritional intake patterns and higher prevalence of obesity than those living in the rest of the United States. This is in line with previous research showing that Mississippi has one of the highest rates of obesity within the US [9]. Second, Mississippi has a significantly larger percentage of rural areas than most other states. 

Future studies should investigate potential pathways that may increase fruit and vegetable intake in all areas of Mississippi, and nationally, within rural areas. Beyond that, research should expand to include examining other behavioral indicators of long-term health (e.g., physical activity). Furthermore, studying interconnections between other variables that may relate to fruit and vegetable intake and rurality are important for future studies. For instance, a recent study [39] has demonstrated a variety of barriers to fruit and vegetable consumption including lack of time, perceived unachievable guidelines, variety of other available foods, high cost, and limited availability of fresh fruits and vegetables. Future studies would benefit from investigating how these various factors may interplay to lead to the decreased consumption of fruits and vegetables and the increased consumption of unhealthy foods such as french fries in more rural areas. Lastly, future studies should include other factors associated with BMI, such as calorie intake and physical activity levels.

## 5. Conclusions

In conclusion, the current study is the first to use nutritional data from the BRFSS to investigate and compare national versus state-level nutritional intake of individuals in Mississippi, demonstrating a potential disconnect at these two levels of scale, thus highlighting the importance of state and local level analyses. Additionally, the findings highlight the need for targeted intervention techniques that can be developed and implemented on a local level to improve the health of those living in rural areas. Given the current findings, it is imperative that Mississippi recognizes the dire need for outreach and education to improve nutrition and obesity rates among its residents to improve long term health outcomes associated with preventable diet-related chronic diseases.

## Figures and Tables

**Table 1:** BRFSS Nutritional Intake Questions.

“Not including juices: how often did you eat fruit?”
“Not including fruit-flavored drinks or fruit juices with added sugar, how often did you drink 100% fruit juice such as apple or orange juice?”
“How often did you eat a green leafy or lettuce salad, with or without other vegetables?”
“How often did you eat any kind of fried potatoes, including french fries, home fries, or hash browns?”
“How often did you eat any other kind of potatoes, or sweet potatoes, such as baked, boiled, mashed potatoes, or potato salad?”
“Not including lettuce salads and potatoes, how often did you eat other vegetables?”

**Table 2:** Participant Demographics.

Variable	Mississippi		USA ^1^	
	*n*	%	*n*	%
Race and Ethnicity				
White, Non-Hispanic	3239	65.3%	333,927	76.5%
Black, Non-Hispanic	1494	30.1%	34,271	7.9%
Hispanic	50	1.0%	37,028	8.5%
Other Race, Non-Hispanic	92	1.9%	21,984	5.0%
Multiracial, Non-Hispanic	88	1.8%	9088	2.1%
Sex				
Male	2033	40.1%	196,692	44.2%
Female	3041	59.9%	247,966	55.7%
Age Category				
18–24	217	4.3%	26,016	5.9%
25–29	202	4.0%	22,186	5.1%
30–34	239	4.8%	24,560	5.6%
35–39	250	5.0%	26,047	5.9%
40–44	268	5.3%	25,032	5.7%
45–49	311	6.2%	29,823	6.8%
50–54	425	8.5%	36,724	8.4%
55–59	526	10.5%	44,598	10.2%
60–64	603	12.0%	49,371	11.2%
65–69	651	13.0%	49,369	11.2%
70–74	529	10.6%	40,870	9.3%
75–79	385	7.7%	28,884	6.6%
80+	404	8.1%	35,425	8.1%
Income Category				
Less than USD 15,000	722	16.3%	36,958	10.0%
USD 15,000–USD 24,999	920	20.8%	61,037	16.5%
USD 25,000–USD 34,999	512	11.6%	39,239	10.6%
USD 35,000–USD 49,999	612	13.8%	52,536	14.2%
USD 50,000 or More	1656	37.4%	180,739	48.8%
Education Level Obtained				
No High School Degree	547	10.8%	32,140	7.2%
Graduated High School	1473	29.0%	121,104	27.2%
Attended College or Technical School	1425	28.1%	123,230	27.7%
Graduated College or Technical School	1611	31.7%	166,779	37.5%
Rurality Level				
In the Center City of an MSA	284	11.3%	59,043	31.2%
Outside the Center City of an MSA but inside the County Containing the Center City	174	6.9%	34,526	18.2%
Inside a Suburban County of the MSA	324	12.9%	34,921	18.5%
Not in an MSA	1731	68.9%	60,753	32.1%

^1^ USA includes data from participants who reported living in the United States of America in states excluding Mississippi.

**Table 3:** Descriptive Statistics and One-Way ANCOVA Results Comparing Mississippi to the Rest of the Nation, Adjusting for Age, Income, Race, Education, and Presence of Children in the Home.

Variable	Mississippi		Rest of U.S.A.		
	Mean	SD	Mean	SD	*F*
Fruit Juice	91.28	90.914	120.93	155.204	31.591 ***
Dark Green Veg.	48.13	52.880	62.13	140.183	7.800 **
Other Veg.	93.08	86.370	105.60	198.755	4.133 *
Total Fruits	131.64	127.701	153.57	185.561	11.830 **
Total Vegetables	192.54	125.856	214.72	294.559	5.472 *
French Fries	29.14	44.018	24.04	54.201	7.755 **
Potatoes	22.78	23.755	23.69	46.029	1.481
Computed BMI	2994.14	793.262	2855.79	694.148	34.963 ***

Note. All analyses controlled for age, income, race, education, and the presence of children in the home. Degrees of freedom for all ANCOVA numerators were 1 and denominators ranged between 93,051 and 95,395, depending on missing data. *** *p* < 0.001. ** *p* < 0.01. * *p* < 0.05.

## Data Availability

Data analyzed for this research are publicly available and can be accessed via the following link: https://www.cdc.gov/brfss/annual_data/annual_2017.html (accessed on 15 December 2020).
